# Viral Strain-Specific Activation of Pathogen-Associated Molecular Pattern-Triggered Immunity Enhances Symptom Severity in Broad Bean Wilt Virus 2 Infection

**DOI:** 10.3389/fpls.2021.746543

**Published:** 2021-09-21

**Authors:** Soo-Jung Han, Boram Choi, Myung-Hwi Kim, Sun-Jung Kwon, Hae-Ryun Kwak, Jang-Kyun Seo

**Affiliations:** ^1^ Department of International Agricultural Technology, Seoul National University, Pyeongchang, South Korea; ^2^ Institute of Green Bio Science and Technology, Seoul National University, Pyeongchang, South Korea; ^3^ Integrated Major in Global Smart Farm, Seoul National University, Seoul, South Korea; ^4^ Crop Protection Division, Rural Development Administration, National Institute of Agricultural Sciences, Wanju, South Korea

**Keywords:** broad bean wilt virus 2, symptom severity, PAMP-triggered immunity, ethylene, transcriptome analysis

## Abstract

Broad bean wilt virus 2 (BBWV2) is an emerging virus in various economically important crops, especially pepper (*Capsicum annuum* L.), worldwide. Recently, the emergence of various BBWV2 strains that induce severe symptoms has increased damage to pepper crops. While the symptomatic variations among virus strains should be associated with differences in the transcriptomic reprogramming of host plants upon infection, underlying molecular mechanisms and associated genes are largely unknown. In the present study, we employed transcriptome analysis to identify responsible host factors for symptom enhancement in the BBWV2-pepper pathosystem using two distinct BBWV2 strains, PAP1 (a severe strain) and RP1 (a mild strain). Comparative analysis of the differentially expressed genes (DEGs) revealed that various genes associated with pathogen-associated molecular pattern (PAMP)-triggered immunity (PTI) and ethylene signaling were significantly upregulated upon infection with the severe PAP1 strain, but not with the mild RP1 strain. Indeed, hormone analysis revealed that ethylene emission was significantly increased in pepper plants infected with PAP1. These observations imply that the activation of the PTI-associated defense responses reinforce symptom formation during BBWV2 infection in a virus strain-specific manner.

## Introduction

The symptom severity of crop diseases is closely related to productivity (Gaunt, 1995). Plant viruses are obligate intracellular parasites that disturb the normal activity of host cells, resulting in a wide range of symptoms. Different strains of plant viruses often induce symptoms with different severity in the host plants. Such symptomatic variation can be triggered by molecular interactions between the host and viral strain-specific factors, leading to transcriptome reprogramming of numerous genes involved not only in defense responses, but also in plant development, physiology, and metabolism (Seo et al., 2018). In this regard, the identification of crucial host genes associated with symptomatic variation and their underlying molecular mechanisms can provide new insights into molecular plant physiological phenomena as well as symptom development.

Pepper (*Capsicum annuum* L.) is an economically important vegetable crop worldwide. However, commercial pepper production in many global areas continues to suffer from various viral diseases. While more than 100 viruses infect pepper plants worldwide (Kenyon et al., 2014), broad bean wilt virus 2 (BBWV2; genus *Fabavirus*, family *Secoviridae*) is an emerging virus in many global areas (Kwak et al., 2013a,b; Ferriol et al., 2014). The incidence of BBWV2 in pepper has increased rapidly over the past decade, because the virus can be easily transmitted by aphids and infect a wide range of plants, including various weed hosts that inhabit areas near pepper fields (Kwak et al., 2013a; Kim et al., 2014a). The BBWV2 genome is composed of two segments of positive-sense single-stranded RNAs, RNA1 (~5.8 knt) and RNA2 (~3.3 knt; Ferrer et al., 2011). Each RNA segment encodes a single open reading frame (ORF) that translates into a large polyprotein precursor. Proteolysis of the polyprotein precursor encoded by RNA1 yields five mature proteins: protease cofactor (Co-Pro), NTP-binding motif (NTBM), VPg, protease (Pro), and RNA-dependent RNA polymerase (RdRp). After the same processing, RNA2 expresses the following three mature proteins: movement protein (MP), large coat protein (LCP), and small coat protein (SCP; Ferrer et al., 2011).

Broad bean wilt virus 2 causes diverse symptoms in pepper, depending on the compatibility between pepper varieties and virus strains, including mild mosaic, malformation, stunting, and chlorosis (Kwak et al., 2013b, 2016). In a previous study, we generated infectious cDNA clones of two pathogenically different BBWV2 strains, namely pBBWV2-RP1 (a mild strain) and pBBWV2-PAP1 (a severe strain; Kwak et al., 2016). Using the chimeric viruses and amino acid substitution mutant viruses derived from pBBWV2-RP1 and pBBWV2-PAP1, we demonstrated that MP is the viral strain-specific factor that determines symptom severity in BBWV2 (Seo et al., 2017). In the present study, we employed comparative transcriptome analysis to gain a better understanding of the molecular mechanisms associated with the development of distinct symptoms caused by the two BBWV2 strains in pepper.

## Materials and Methods

### Plant Growth and Virus Inoculation

Pepper (*Capsicum annuum* L. cv. Sinhong) and *Nicotiana benthamiana* plants were grown in an insect-free growth chamber in a cycle of 16h light at 26°C and 8h darkness at 24°C. Full-length infectious cDNA clones of two BBWV2 strains (RP1 and PAP1), as described in our previous study (Kwak et al., 2016), were used as the viral sources for each strain. Infectious cDNA clones of the virus strains were inoculated into the leaves of 2-week-old *N. benthamiana* plants by *Agrobacterium*-mediated infiltration (agroinfiltration), as previously described (Seo et al., 2009). Crude sap prepared from the symptomatic leaves of *N. benthamiana* infected with each virus strain was subsequently used for mechanical inoculation of pepper. Crude sap was rubbed onto leaves of 3-week-old pepper plants dusted with carborundum (400 mesh). After inoculation, the leaves were washed with sterile water.

### Sample Preparation, Library Construction, RNA Sequencing, and Virus Detection

About 2weeks after mechanical inoculation, symptomatic upper leaf samples were collected from nine individual plants infected with BBWV2-RP1 or PAP1, and frozen immediately in liquid nitrogen before use. Leaf samples from three individual plants were pooled together for RNA isolation. Thus, three biological replicate RNA samples were obtained for each experimental group. Similarly, leaf samples from uninoculated plants were used as healthy controls. Total RNA was extracted using the PureLink® RNA Mini Kit (Ambion, United States) and subjected to library construction using the Illumina TruSeq RNA Sample Preparation Kit v2 (Illumina, Inc., United States). Nine libraries were constructed and quantified with the KAPA library quantification kit (Kapa Biosystems, United States). Sequencing on an Illumina HiSeq2000 sequencer (Illumina, Inc., United States) was performed by TheragenEtex Inc. (Suwon, South Korea). To analyze virus accumulation levels in the systemically infected leaves, the same RNA preparations used for the RNA sequencing were subjected to quantitative real-time RT-PCR (qRT-PCR) using an iCycler iQ5 qRT-PCR detection system (Bio-Rad, United States) with the following specific primers: BBWV2-R1-RT-Fw (5'-TCACAGGTTATGCCGCTTGT-3') and BBWV2-R1-RT-Rv (5'-TCACTCGTCCCAAGCTGTTC-3') for BBWV2 RNA1 detection and UBI2-F (5'-TACCCTTCACCTTGTCCTCC-3') and UBI2-R (5'-GCCATCCTCCAACTGTTTTC-3') for *ubiquitin2* mRNA detection. The *ubiquitin2* gene (*CA.*PGAv.1.6.scaffold337.91) was used as an internal reference to standardize the different samples. Three biological and technical replicates were used per sample.

### Processing of mRNA Sequence Data

The Illumina pipeline filtrated raw sequence reads, which were then mapped to the reference transcripts of *C. annuum* cv. CM334 (Kim et al., 2014b) retrieved from the Pepper Genome Platform^1^ using the RNA-seq mapping algorithm implemented in bowtie2 (v2.1.0) software (Langmead et al., 2009), allowing all aligning with a maximum of two mismatches. The raw data were deposited in the National Center for Biotechnology Information (NCBI) SRA database with BioProject accession number PRJNA751625. To eliminate bias caused by variations in sequencing depth, the number of mapped clean reads for each genes was measured and then normalized with the DESeq package in R software (Anders and Huber, 2010). Differentially expressed genes (DEGs) were identified by a ≤2-fold change in read coverage and a binomial test with a false discovery rate (FDR)≤0.01. The FDR was applied to identify the threshold value of *p* for multiple tests and was calculated using DESeq. Correlation analysis and hierarchical clustering were performed to categorize the genes according to patterns of expression using the AMAP library in R (Lucas, 2014).

### Gene Enrichment Analysis

Gene ontology (GO) analysis was performed to functionally annotate the DEGs based on the protein sequence similarity (e-value cutoff≤1e-10) in the GO database (Ashburner et al., 2000). The number of DEGs assigned in each GO term was counted using the in-house scripts of SEEDERS Inc (Daejeon, South Korea). GO term enrichment was performed using the PANTHER overrepresentation test (Mi et al., 2013). Functional enrichment analysis was performed to assign biological relevance to the gene network modules using agriGO v2.0 (Tian et al., 2017). The Kyoto Encyclopedia of Genes and Genomes (KEGG) pathway was analyzed using the sequence similarity (e-value cut off≤1e-10, identity ≥90) of pepper proteins in the KEGG database (Kanehisa and Goto, 2000).

### DAB Staining

3,3'-diaminobenzidine (DAB) staining was performed to detect reactive oxygen species (ROS) as previously described (Wang et al., 2017). Briefly, upper uninoculated leaf samples were collected from the virus-infected and healthy plants at 14days post-inoculation (dpi). The leaf samples were stained with 1mg/ml DAB (Sigma, United States) by vacuum infiltration and incubated for 8h at room temperature in the dark. The leaf samples were then destained with 90% ethanol. The destained samples were mounted in 50% glycerol and observed by light microscopy.

### Ethylene Analysis

Ethylene production from virus-infected and healthy plants was measured at 14dpi as described previously with minor modifications (Hou et al., 2018). Briefly, 5g of the upper uninoculated leaves were sampled from three plants and placed in 400ml glass jars for 5h at 26°C in the dark. Next, 1ml of the jar headspace gas sample was sampled and analyzed using a gas chromatograph equipped with a hydrogen flame ionization detector and an activated alumina column (Agilent Technologies, United States). Samples were compared to a standard of known concentration. The experiment was repeated three times, and data were averaged.

### Engineering of the BBWV2 Viral Construct

The coding region of the *ethylene response factor 5* gene (*ERF5*; Gene ID: *CA.*PGAv.1.6.scaffold296.14) was amplified by RT-PCR using a primer pair (5'-GAAGATCTATGGATACTTCTTCACTAGAT-3' and 5'-GATCCTAGGTGAAACCAAAAGTTGAGAAAAACCA-3'; *Bgl*II and *Avr*II sites are shown in bold). The amplified fragments were digested with *Bgl*II and *Avr*II and cloned into pBBWV2-R2-OE (Choi et al., 2019), which was opened with *Bgl*II and *Avr*II. The resulting construct was named pBBWV2-RP1-R2-ERF5. For the agroinfiltration of pBBWV2-RP1-R2-ERF5, the plasmid DNA was first transformed into *Agrobacterium tumefaciens* strain EHA105, and agroinfiltration was subsequently performed as previously described (Choi et al., 2019).

### Quantitative Real-Time PCR Validation

To validate the RNA sequencing data, the expression levels of *basic pathogenesis-related protein 1* gene (*CA.*PGAv.1.6.scaffold608.26), *ripening-related protein grip22* gene (*CA.*PGAv.1.6.scaffold631.48), *cysteine-rich receptor-like protein kinase 25* gene (*CA.*PGAv.1.6.scaffold674.24), *glycine-rich protein* gene (*CA.*PGAv.1.6.scaffold1405.6), *pathogenesis-related protein 3* gene (*CA.*PGAv.1.6.scaffold890.65), *WRKY transcription factor 70* gene (*CA.*PGAv.1.6.scaffold788.4), *ACC oxidase* gene (*CA.*PGAv.1.6.scaffold793.11), *LRR receptor-like serine/threonine-protein kinase* gene (*CA.*PGAv.1.6.scaffold1537.4), *ethylene responsive factor 5* gene (*CA.*PGAv.1.6.scaffold296.14), *chitin-binding lectin 1-like* gene (*CA.*PGAv.1.6.scaffold837.4), *ABC transporter B family member 11* gene (*CA.*PGAv.1.6.scaffold484.97), and *mitogen-activated protein kinase* gene (*CA.*PGAv.1.6.scaffold200.11) were analyzed using qRT-PCR. The *ubiquitin2* gene (*CA.*PGAv.1.6.scaffold337.91) was selected as an internal reference for the qRT-PCR experiments because this gene showed stable expression patterns across all treatments in the RNA sequencing results (data not shown). cDNA was synthesized from the same RNA preparations (DNase-treated) used for the RNA sequencing using Superscript III (Invitrogen, United States) with oligo dT primers. The resulting cDNA was subjected to qRT-PCR using an iCycler iQ5 qRT-PCR detection system (Bio-Rad, United States) with specific primers (listed in Supplementary Table S1). Three biological and three technical replicates were used per sample. qRT-PCR data were calculated as log2 fold changes and compared with log2 fold values obtained from the RNA sequencing.

## Results

### Transcriptome Analysis of Symptomatic Variation Caused by Two Distinct BBWV2 Strains in Pepper

**Figure 1:**
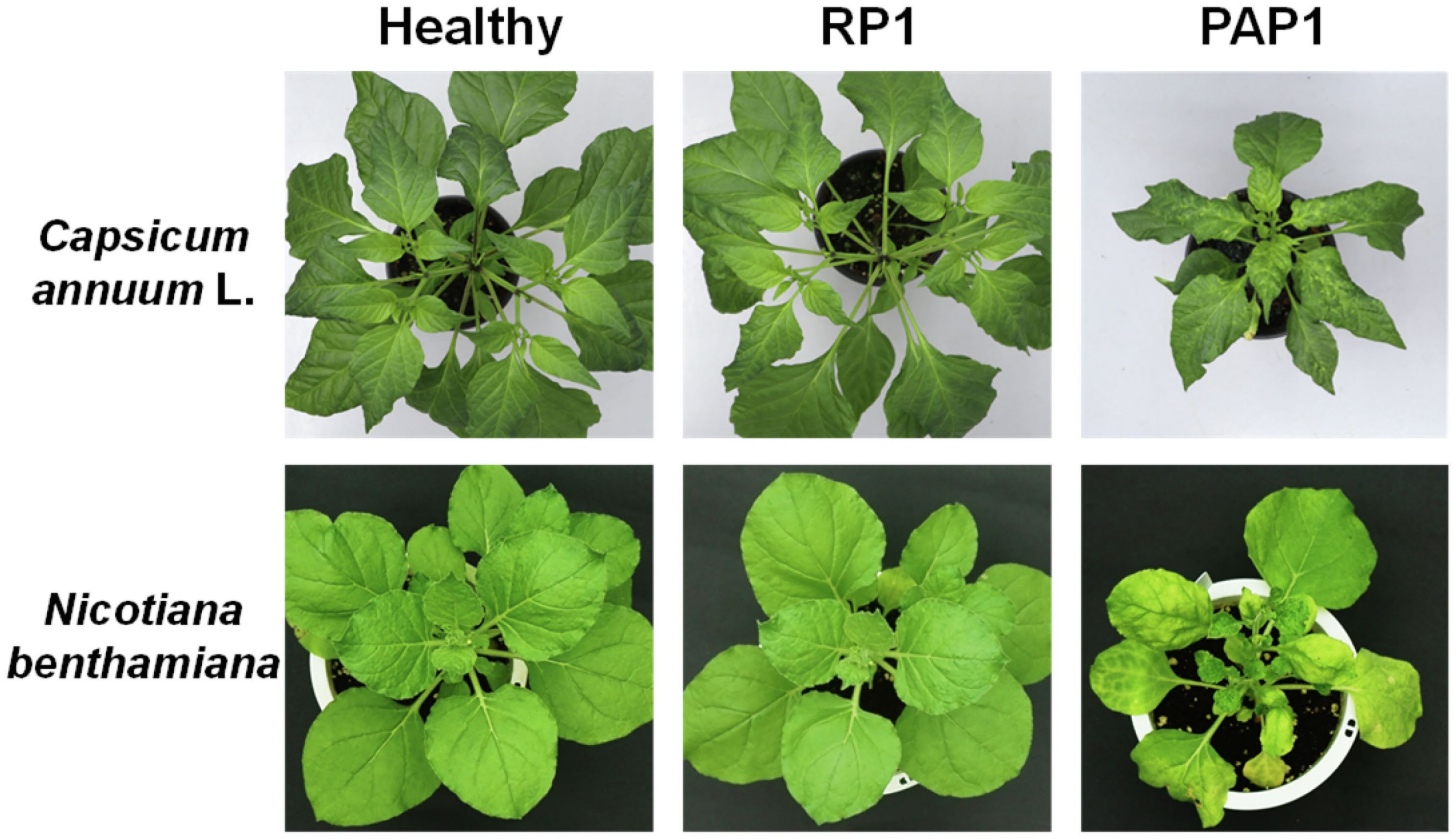
Symptomatic responses of pepper (*Capsicum annuum* L.) and *Nicotiana benthamiana* plants upon infection with BBWW2 isolates PAP1 or RP1. BBWV2-RP1 induced no visible symptoms in pepper and very mild symptoms in *N. benthamiana*, whereas BBWV2-PAP1 caused severe disease symptoms in pepper and *N. benthamiana*.

Two distinct BBWV2 strains, PAP1 and RP1, cause symptoms with different severity in pepper and *N. benthamiana*: BBWV2-PAP1 induced severe symptoms of mosaic, leaf malformation, and stunting in pepper and *N. benthamiana*, whereas BBWV2-RP1 caused no visible symptoms in pepper and very mild symptoms in *N. benthamiana* (Figure 1).

**Figure 2:**
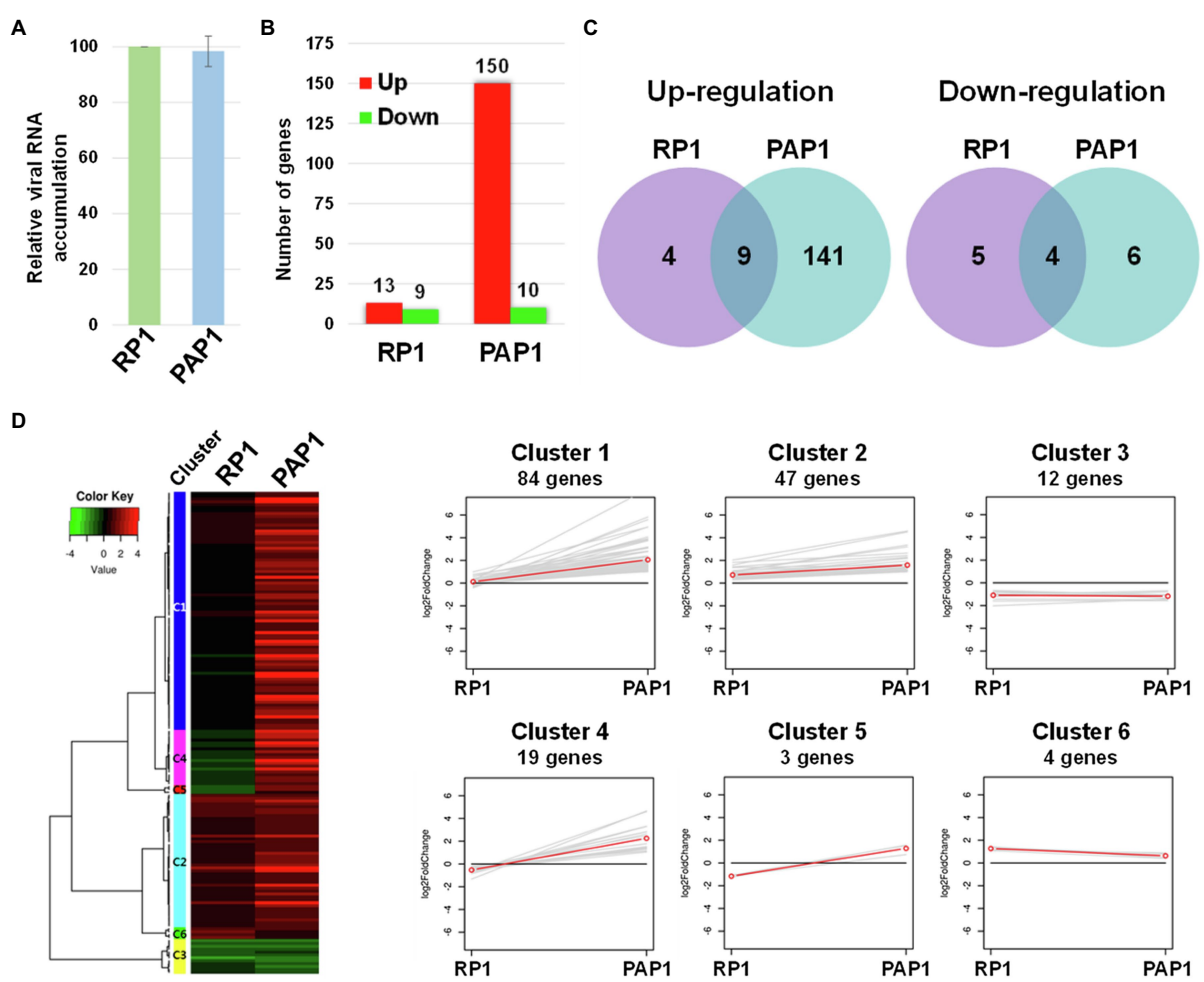
Transcriptome analysis of distinct symptoms induced by two different BBWV2 strains in pepper. **(A)** Relative accumulation analysis of BBWV2-PAP1 and RP1 in pepper by quantitative real-time RT-PCR (qRT-PCR). Means±SD from three independent experiments are shown and each column represents one group with nine plants; statistical analysis was performed using paired Student’s *t*-tests to detect significant differences. **(B)** The number of DEGs identified by RNA sequencing of the transcriptomes in response to infection with BBWV2-PAP1 or RP1. The DEGs were identified by comparing the virus-infected samples to healthy samples using a 2-fold change in expression with a false discovery rate (FDR) of ≤0.01. Red and green bars indicate the numbers of up and downregulated DEGs, respectively. **(C)** Venn diagrams display the number of up or downregulated DEGs upon infection with each virus strain. **(D)** Hierarchical clustering of the identified DEGs. The heat-map was produced using a log scale of the fragments per kilobase of exon per million fragments mapped (FPKM) data obtained by RNA Sequencing. Red and green indicate up and downregulation, respectively. The linear graphs show the expression patterns of each cluster.

We sought to examine host genes associated with viral strain-specific symptoms in the BBWV2-pepper pathosystem. To this end, Illumina RNA sequencing was performed to examine the transcriptomic changes in pepper upon infection with BBWV2-PAP1 and RP1. We first analyzed viral RNA accumulation of each strain in the systemic leaves using qRT-PCR. The results showed no significant difference in viral RNA accumulation between the two BBWV2 strains (Figure 2A), suggesting that the symptomatic difference between the two BBWV2 strains was not due to differences in virus accumulation levels. The same RNA preparation was subjected to cDNA library construction. Nine cDNA libraries (three for each sample) were sequenced by Illumina RNA sequencing. For mapping, we used a total of 35,884 reference transcripts of *C. annuum* cv. CM334 (Kim et al., 2014b). The Illumina pipeline filtered and trimmed the raw HiSeq reads, yielding approximately 81–86 million clean pair-end reads from each of the nine libraries (Table 1). The acquired reads were mapped on the reference pepper transcripts; this resulted in the mapping of approximately 83.6–92.81% of the nucleotides (Table 1). The nucleotide coverage for healthy, BBWV2-RP1, and BBWV2-PAP1 was 175, 164.68, and 168.45 times, respectively (Table 1).

**Table 1:** Read statistics for RNA sequencing of pepper plants infected with broad bean wilt virus 2 (BBWV2)-RP1 or PAP1.

Index	Healthy	BBWV2-RP1	BBWV2-PAP1
No. of trimmed reads (%)	80,988,234 (90.73%)	83,192,654 (91.68%)	85,958,586 (91.83%)
No. of mapped reads (%)	75,175,224 (92.82%)	70,481,206 (84.72%)	71,890,360 (83.63%)
No. of mapped nucleotides (%)	6,848,712,056 (92.81%)	6,444,797,133 (84.66%)	6,592,273,522 (83.62%)
Average coverage	175.00	164.68	168.45

### Identification of DEGs in Response to Infection With BBWV2-PAP1 or RP1

The fragments per kilobase of exon per million fragments mapped (FPKM) value was used to normalize the expression levels of the mapped genes. DEGs were identified by statistically comparing the FPKM values of two samples using a 2-fold change in expression with the FDR≤0.01. By comparing virus-infected samples to healthy samples, we identified 160 and 22 DEGs in response to infection with BBWV2-PAP1 and RP1, respectively (Figure 2B; Supplementary Table S2). When the DEGs were compared between the two infection conditions, a total of 147 genes were identified to be specifically expressed in response to BBWV2-PAP1 (upregulation of 141 genes and downregulation of six genes; Figure 2C; Table 2; Supplementary Table S2). Comparatively few genes were specifically affected upon infection with BBWV2-RP1 (upregulation of four genes and downregulation of five genes; Figure 2C; Supplementary Table S2). Hierarchical clustering of the total DEGs revealed six distinct groups based on the expression patterns in each infection condition (Figure 2D). For example, cluster 4 contained 19 genes upregulated by BBWV2-PAP1 infection but downregulated by BBWV2-RP1 infection.

**Table 2:** Top 30 upregulated differentially expressed genes (DEGs) upon infection with BBWV2-PAP1.

Gene ID	Seq. description	log2 fold change		*Arabidosis* homologous
		PAP1	RP1	
*CA.*PGAv.1.6.scaffold608.26	Basic pathogenesis-related protein 1	8.29	0.24	AT2G14580.1
*CA.*PGAv.1.6.scaffold631.48	Ripening-related protein grip22	5.82	−0.34	NA
*CA.*PGAv.1.6.scaffold674.24	Cysteine-rich receptor-like protein kinase 25	5.59	−0.15	AT4G05200.1
*CA.*PGAv.1.6.scaffold58.30	Nucleoside triphosphate hydrolases superfamily protein	4.95	−0.41	AT3G28580.1
*CA.*PGAv.1.6.scaffold1405.6	Glycine-rich protein	4.94	0.98	NA
*CA.*PGAv.1.6.scaffold890.65	Pathogenesis-related protein 3	4.60	−0.69	AT3G12500.1
*CA.*PGAv.1.6.scaffold575.21	CER1-like 1	4.59	2.03	AT1G02205.1
*CA.*PGAv.1.6.scaffold79.50	Glutathione S-transferase parC	4.50	1.59	AT1G78380.1
*CA.*PGAv.1.6.scaffold788.4	WRKY transcription factor 70	4.31	−0.79	AT3G56400.1
*CA.*PGAv.1.6.scaffold206.16	Pathogenesis-related protein 2	4.17	0.06	AT3G57260.1
*CA.*PGAv.1.6.scaffold793.11	ACC oxidase	4.07	−0.05	AT1G06620.1
*CA.*PGAv.1.6.scaffold647.6	LRR receptor-like serine/threonine-protein kinase GSO2-like	4.03	−0.42	AT5G46330.1
*CA.*PGAv.1.6.scaffold753.4	HYPER-SENSITIVITY-RELATED 4-like	3.92	0.60	AT3G50930.1
*CA.*PGAv.1.6.scaffold628.31	ABC transporter A family member 3	3.90	0.24	AT5G61700.1
*CA.*PGAv.1.6.scaffold1537.4	LRR receptor-like serine/threonine-protein kinase	3.82	0.17	AT3G47570.1
*CA.*PGAv.1.6.scaffold823.8	Ammonium transporter 2 member 2	3.73	−0.17	AT2G38290.1
*CA.*PGAv.1.6.scaffold242.12	Uncharacterized isomerase BH0283-like	3.35	1.05	AT4G02860.1
*CA.*PGAv.1.6.scaffold1134.9	Phosphoglycerate mutase-like	3.32	−0.61	AT3G05170.1
*CA.*PGAv.1.6.scaffold577.9	Cytochrome P450 CYP72A219-like	3.20	1.03	AT3G14690.1
*CA.*PGAv.1.6.scaffold628.32	ABC transporter A family member 2	3.19	0.09	AT3G47730.1
*CA.*PGAv.1.6.scaffold608.6	LRR receptor-like serine/threonine-protein kinase GSO1-like	3.13	0.76	AT1G71390.1
*CA.*PGAv.1.6.scaffold1592.1	COBRA-like protein 5	3.10	−0.10	AT5G15630.1
*CA.*PGAv.1.6.scaffold1110.29	G-type lectin S-receptor-like serine/threonine-protein kinase	3.08	0.40	AT4G27290.1
*CA.*PGAv.1.6.scaffold702.22	Senescence-specific cysteine protease SAG12	2.83	−0.39	AT5G45890.1
*CA.*PGAv.1.6.scaffold1152.14	Linoleate 9S-lipoxygenase 5-like	2.82	0.76	AT1G55020.1
*CA.*PGAv.1.6.scaffold702.24	Senescence-specific cysteine protease SAG39-like	2.82	−0.46	AT5G45890.1
*CA.*PGAv.1.6.scaffold954.24	AAA-ATPase-like	2.81	−0.19	AT3G28580.1
*CA.*PGAv.1.6.scaffold27.14	NRT1/PTR FAMILY 2.13-like	2.80	−0.13	AT1G69870.1
*CA.*PGAv.1.6.scaffold1218.1	Lipid transfer protein EARLI 1-like	2.77	0.22	AT4G12480.1
*CA.*PGAv.1.6.scaffold2870.1	LRR receptor-like protein kinase	2.64	−0.72	AT4G08850.1

### Gene Ontology Analysis of Identified DEGs

**Figure 3:**
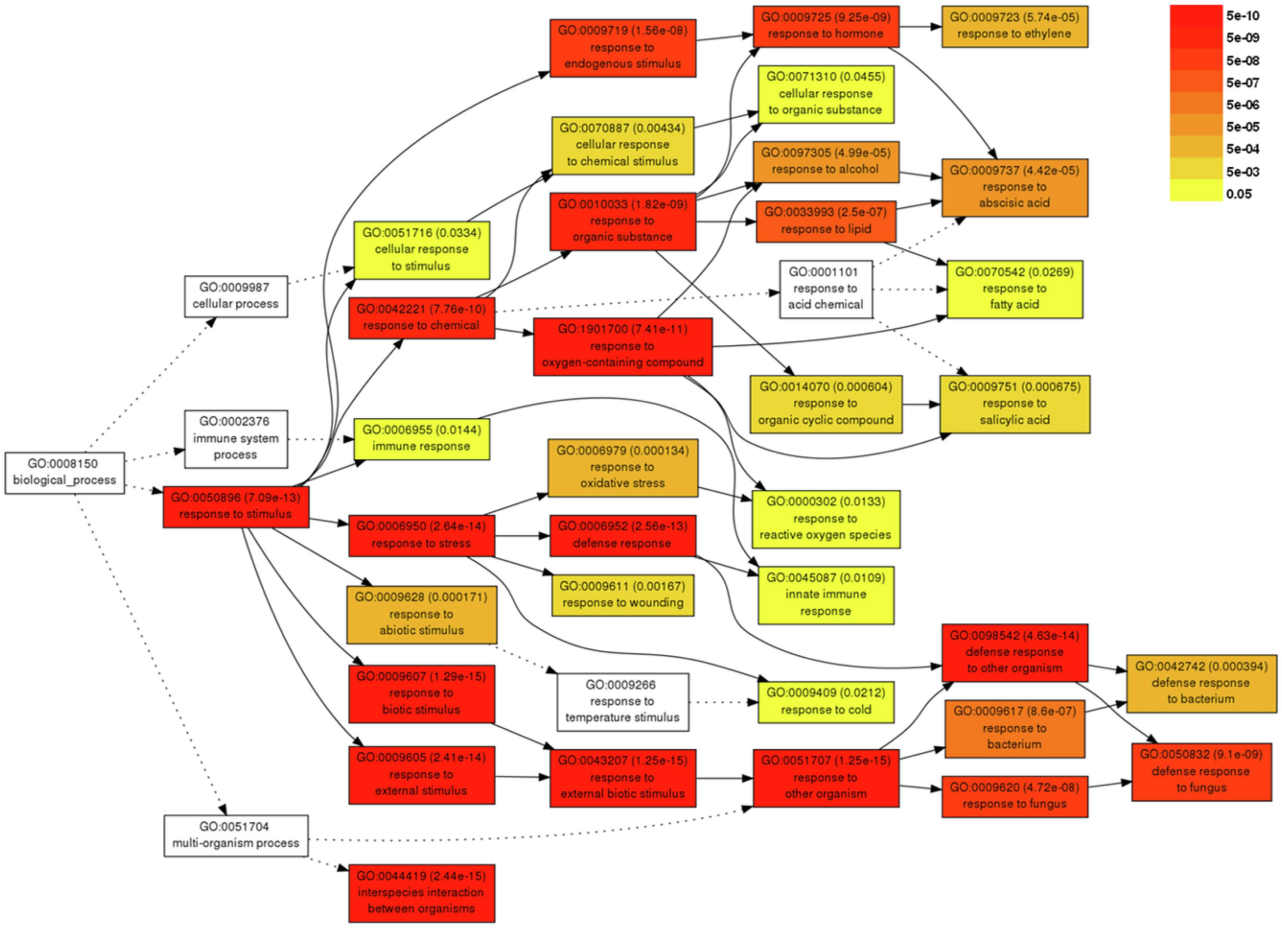
Hierarchical tree graph of overrepresented gene ontology (GO) terms in response to BBWV2-PAP1. The graph was produced by singular enrichment analysis (SEA) using agriGO. Boxes in the graph represent GO terms labeled by GO number, statistical information, and term definition. The degree of color saturation in a box is proportional to the enrichment level of the term.

For a better understanding of the DEGs associated with severe symptom development caused by BBWV2-PAP1, GO analysis was performed using the DEGs upregulated upon BBWV2-PAP1 infection. A total of 37 GO terms were significantly enriched by infection with BBWV2-PAP1 (Supplementary Table S3). Most BBWV2-PAP1-specific GO terms were associated with defense response and response to stimulus, e.g., systemic acquired resistance (GO:0009627), response to ethylene (GO:0009723), defense response to fungus (GO:0050832), response to salicylic acid (GO:0009751), innate immune response (GO:0045087), response to reactive oxygen species (GO:0000302), and immune response (GO:0006955). Hierarchical GO enrichment analysis using agriGO v2.0 further showed that the identified GO terms were highly correlated in a network context (Figure 3). The analysis also highlighted a few downstream GO terms, including defense response to fungus (GO:0050832), response to hormone (GO:0009725), and response to ethylene (GO:0009723), which are likely directly correlated to symptom development in response to BBWV2-PAP1 (Figure 3).

### Important KEGG Pathways Influenced by BBWV2-PAP1 Infection

**Figure 4:**
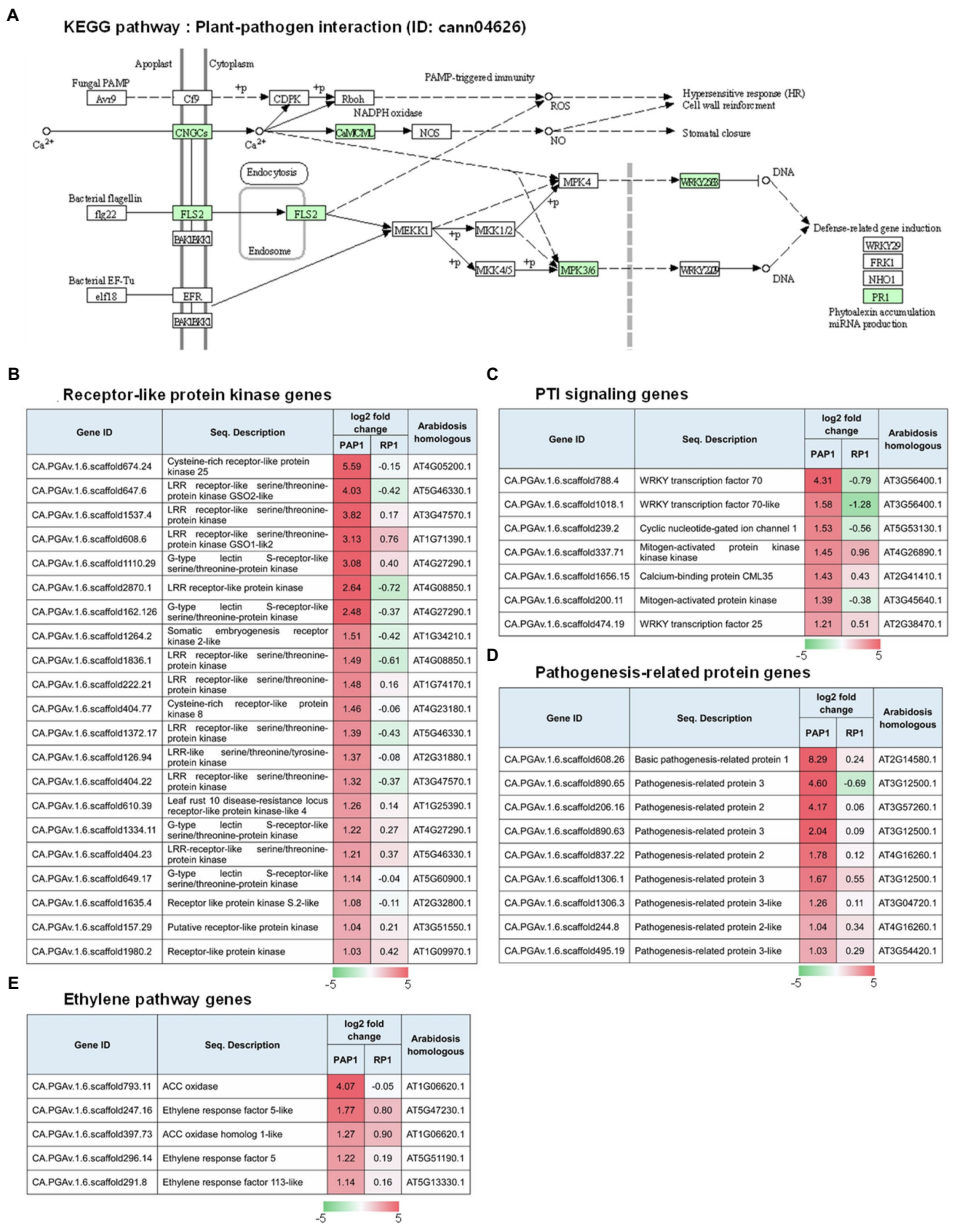
Regulation of the genes involved in pathogen-associated molecular pattern (PAMP)-triggered immunity (PTI) upon BBWV2-PAP1 infection in pepper. **(A)** Kyoto Encyclopedia of Genes and Genomes (KEGG) pathway enrichment analysis. The DEGs upregulated upon BBWV2-PAP1 infection were mapped to the plant–pathogen interaction KEGG pathway (ID: cann04626). Green boxes represent genes upregulated upon BBWV2-PAP1 infection. **(B)** Receptor-like protein kinase genes upregulated upon BBWV2-PAP1 infection. **(C)** DEGs associated with PTI signaling. **(D)** Pathogenesis-related protein genes upregulated upon BBWV2-PAP1 infection. **(E)** DEGs associated with the ethylene pathway. The changes in gene expression were calculated using a log scale of the FPKM data obtained by RNA Sequencing. Red and green indicate up and downregulation, respectively.

Kyoto encyclopedia of genes and genomes pathway analysis was performed to understand the relationship between DEGs and the associated pathways. KEGG analysis using the DEGs upregulated in response to BBWV2-PAP1 infection identified some of the highly ranked pathways, including plant–pathogen interaction, metabolic pathways, plant hormone signal transduction, and pathways related to plant pigment metabolism (i.e., flavonoid biosynthesis, flavone and flavonol biosynthesis, and biosynthesis of secondary metabolites; Table 3). In particular, the most significant enrichment of upregulated DEGs was found in the KEGG pathway of plant–pathogen interaction (Pathway ID: cann04626; Table 3; Figure 4A). Various receptor-like protein kinase genes (Figure 4B), pathogen-associated molecular pattern (PAMP)-triggered immunity (PTI) signaling genes (Figure 4C), and pathogenesis-related protein genes (Figure 4D) involved in the KEGG pathway of plant–pathogen interaction were significantly upregulated in response to infection with BBWV2-PAP1. KEGG analysis also identified a few ethylene responsive transcription factor genes, including *ERF5*, in the plant hormone signal transduction pathway (Pathway ID: cann00943; Figure 4E). Our DEG analysis also revealed that key genes involved in ethylene biosynthesis (i.e., ACC oxidase genes) were significantly upregulated in response to infection with BBWV2-PAP1 (Figure 4E). Overall, our results revealed that the PTI and ethylene pathways were activated in BBWV2-PAP1-infectged pepper.

**Table 3:** Top 10 Kyoto Encyclopedia of Genes and Genomes (KEGG) pathways enriched with upregulated DEGs when infected with BBWV2-PAP1.

Pathway	Pathway ID	DEGs	*p*
Plant–pathogen interaction	cann04626	37	0.000013
Metabolic pathways	cann00480	10	0.000386
Plant hormone signal transduction	cann00943	4	0.001894
Flavonoid biosynthesis	cann00905	3	0.007710
Amino sugar and nucleotide sugar metabolism	cann00520	4	0.045420
Biosynthesis of secondary metabolites	cann01040	4	0.045583
Flavone and flavonol biosynthesis	cann01212	3	0.049148
Protein processing in endoplasmic reticulum	cann00514	1	0.161728
Glycerophospholipid metabolism	cann00780	1	0.168753
Arginine and proline metabolism	cann00760	1	0.196282

### BBWV2-PAP1 Infection Increases ROS Accumulation and Ethylene Emission in Pepper

**Figure 5:**
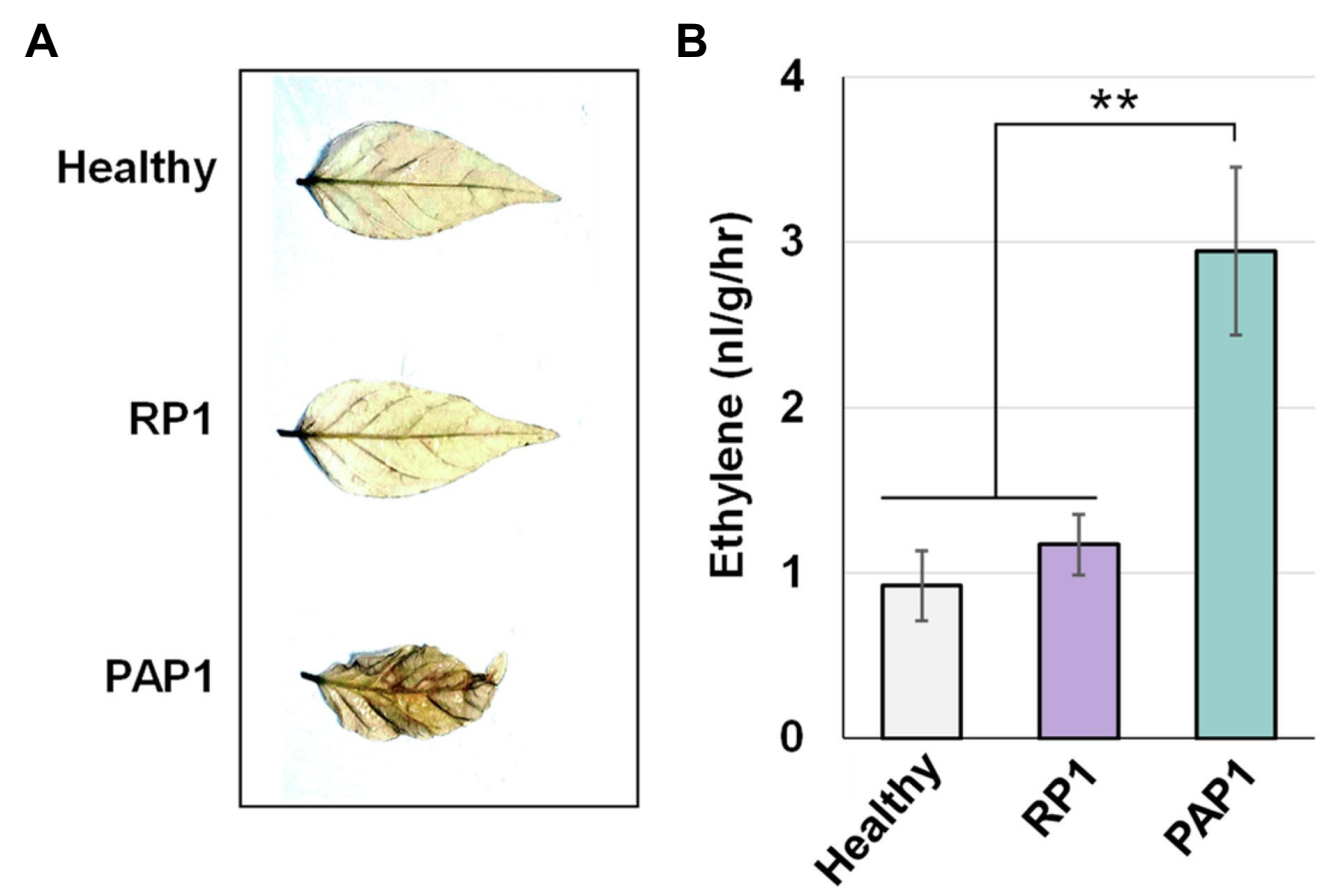
Effect of BBWV2-PAP1 infection on reactive oxygen species (ROS) accumulation and ethylene production in pepper. **(A)** BBWV2-PAP1 infection increased ROS accumulation. Systemic leaves of pepper plants infected with BBWV2-PAP1 or RP1 were analyzed by 3,3'-diaminobenzidine (DAB) staining to visualize ROS accumulation. **(B)** BBWV2-PAP1 infection increased ethylene production. Ethylene emission from the upper uninoculated leaves of virus-infected and healthy plants was measured by gas chromatography. Means±SD from three independent experiments are shown and each column represents one group with nine plants. Statistical analysis was performed using paired Student’s *t*-tests to detect significant differences analyses (^**^ *p*<0.01).

Pathogen-associated molecular pattern-triggered immunity response typically accompanies the accumulation of ROS and is sometimes associated with ethylene pathways (Peng et al., 2018). Because our transcriptomic analysis revealed that BBWV2-PAP1 infection activates PTI and ethylene pathways in pepper, we investigated whether ROS accumulation and ethylene production are enhanced in BBWV2-PAP1-infected leaf tissues. To detect ROS accumulation, DAB staining was performed on systemic leaves of the pepper plants infected with BBWV2-PAP1 or RP1. The leaves infected with BBWV2-PAP1 showed more intense staining than BBWV2-RP1-infected leaves (Figure 5A), indicating that ROS accumulation was specifically elevated by infection with BBWV2-PAP1 but not RP1.

To verify whether activation of the ethylene pathway leads to an increase in ethylene production in BBWV2-PAP1-infected pepper plants, ethylene emission was assessed for detached leaves infected with BBWV2-PAP1 or RP1. There was no significant difference in ethylene production in healthy and BBWV2-RP1-infected leaves (Figure 5B); however, ethylene production was considerably higher in BBWV2-PAP1-infected leaves than in BBWV2-RP1-infected leaves (Figure 5B). This implies that, consistent with the transcriptional upregulation of the ethylene pathway genes, BBWV2-PAP1 infection causes an increase in ethylene production in pepper.

### Overexpression of *Ethylene Responsive Factor 5* Enhances Symptom Severity During BBWV2-RP1 Infection

**Figure 6:**
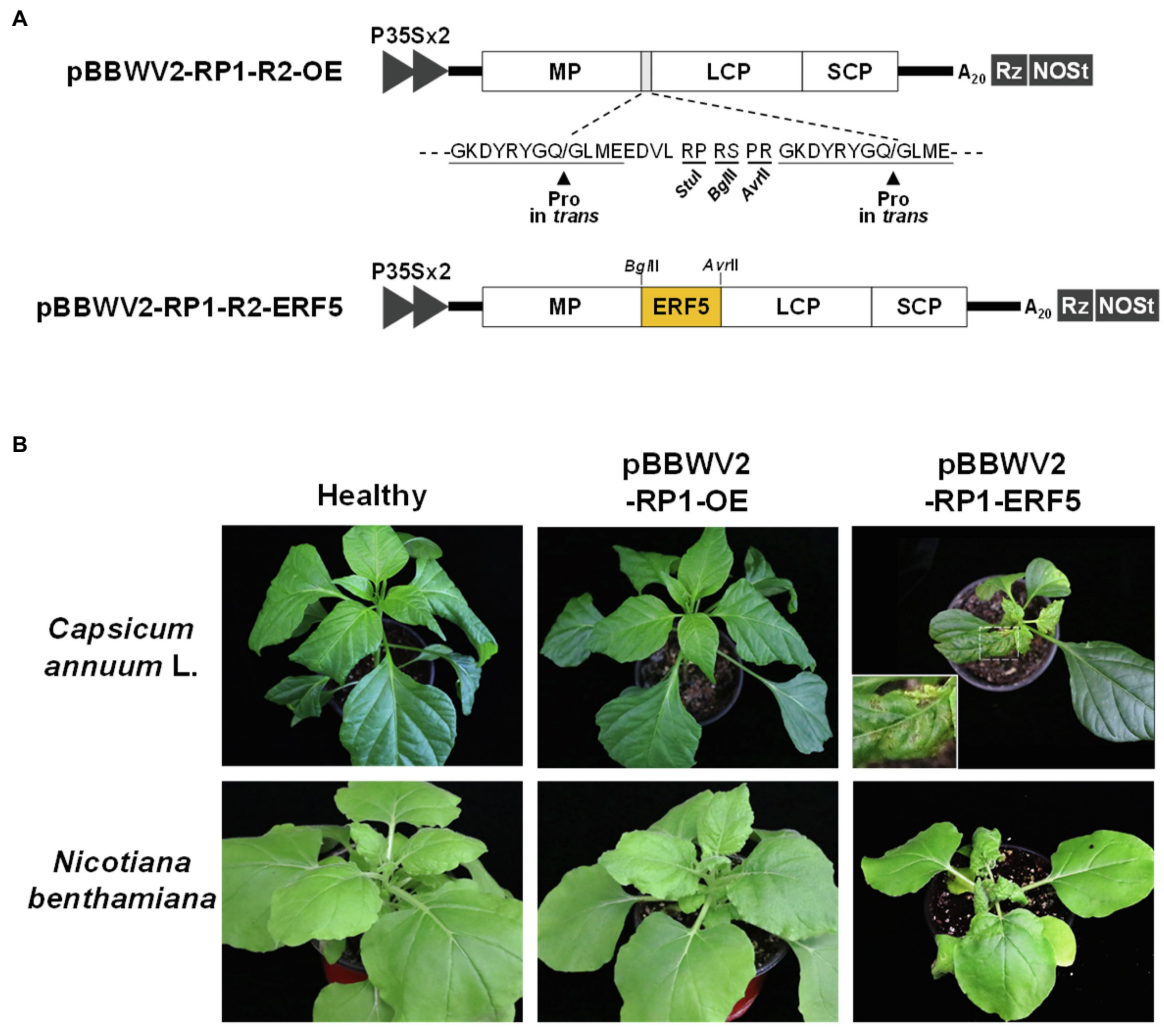
Overexpression of *ethylene responsive factor 5* (*ERF5*) enhances symptom severity in pepper infected with the BBWV2 mild strain RP1. **(A)** Schematic representation of the BBWV2-RP1 RNA2 recombinant constructs. The *ERF5* gene was inserted between movement protein (MP) and large coat protein (LCP) to be expressed by proteolysis when the virus replicates. *In vivo* transcription of the BBWV2-RP1 RNA2 recombinant constructs is under the control of a double 35S promoter (P35SX2), a cis-cleaving ribozyme sequence (Rz), and a NOS terminator (NOSt). **(B)** Symptomatic responses in pepper and *N. benthamiana* plants upon infection with pBBWV2-RP1-ERF5. The inocula are indicated above each image. An enlargement of the boxed area is shown in the upper right panel.

Ethylene biosynthesis is strongly induced in PTI and plays an important role in defense responses (Yang et al., 2017; Peng et al., 2018). ERF5 functions as a positive regulators of ethylene-mediated immunity in *Arabidopsis* (Moffat et al., 2012; Son et al., 2012). Thus, we hypothesized that if the activation of ethylene signaling pathways is correlated with symptom severity in pepper infected with BBWV2, the ectopic overexpression of ERF5 would enhance symptom severity in pepper infected with BBWV2-RP1 (a mild strain). Testing this hypothesis required the overexpression of ERF5 in the pepper cells infected with BBWV2-RP1. For this, we utilized a viral vector system generated by engineering BBWV2-RP1 to overexpress foreign genes in pepper (Choi et al., 2019). Specifically, we engineered the BBWV2-RP1-based vector to overexpress ERF5 in the BBWV2-RP1-infected cells, and the resulting construct was named pBBWV2-RP1-R2-ERF5 (Figure 6A). To examine if the ERF5 overexpression affects symptom severity caused by BBWV2, pepper and *N. benthamiana* plants were infiltrated with a mixture of *Agrobacterium* cultures containing pBBWV2-RP1-R1 and pBBWV2-RP1-R2-ERF5. Interestingly, both plant species were systemically infected with the recombinant BBWV2 vector overexpressing ERF5 and developed severe symptoms of leaf malformation and stunting (Figure 6B). In particular, necrotic cell death responses were also observed in the systemically infected pepper leaves (Figure 6B). Therefore, our results revealed that the ethylene pathway-mediated responses specifically induced by BBWV2-PAP1 are associated with enhanced symptom severity in BBWV2 infection.

### Quantitative Real-Time PCR Validation of RNA Sequencing Data

To validate the gene expression data from the RNA sequencing, the expression levels of 12 representative genes that significantly altered in response to virus infection were evaluated by qRT-PCR using the same RNA preparations used for RNA sequencing. The expression of target genes was normalized to that of the *ubiquitin2* gene (*CA.*PGAv.1.6.scaffold337.91) as an internal control. For each gene, relative expression levels were calculated and compared to the RNA sequencing results. In general, the qRT-PCR results for all of the tested genes were consistent with the RNA sequencing results (Supplementary Table S4). This demonstrates that the changes in gene expression determined by the RNA sequencing were accurate.

## Discussion

Broad bean wilt virus 2 is an emerging virus in various economically important crops worldwide. BBWV2 has a wide host range, and the viral strains isolated from different hosts are genetically and pathogenically differentiated (Ferrer et al., 2011; Kwak et al., 2013a, 2016). Symptomatic variation among different virus strains in a host plant is primarily due to strain-specific viral proteins. Strain-specific viral proteins can have different activities to their original functions or interact with other proteins. In BBWV2, MP was identified as a strain-specific elicitor that determine symptom severity in pepper and *N. benthamiana* (Kwak et al., 2016; Seo et al., 2017); however, the molecular mechanisms and host genes involved in symptom development upon BBWV2 infection remain unknown.

Different symptoms induced by two different strains of BBWV2 (PAP1 and RP1) indicate that transcriptional responses must be different. Notably, BBWV2-RP1 induced no visible symptoms in pepper (Figure 1) while its replication level was similar to BBWV2-PAP1 (Figure 2A). Indeed, only a small number of DEGs were identified in pepper infected with BBWV2-RP1 (Figures 2B,C). On the other hand, BBWV2-PAP1 infection resulted in the upregulation of 150 DEGs (Figure 2B). Our GO term and KEGG pathway analyses revealed that many of the upregulated DEGs were PTI-associated genes, including receptor-like protein kinase genes, PTI signaling genes, and pathogenesis-related protein genes (Figures 3, 4).

Plant cells can recognize microbial invasions through the perception of PAMPs by pattern-recognition receptors (PRRs) localized on the plasma membrane, triggering the PTI activation (Peng et al., 2018). PTI involves a series of downstream defense responses, such as mitogen-activated protein kinase activation, ROS accumulation, plant hormone biosynthesis, and expression of pathogenesis-related proteins, resulting in the prevention of pathogen colonization (Peng et al., 2018). Our comparative transcriptome analysis revealed that PTI pathways can be activated in a virus strain-specific manner in pepper infected with BBWV2 (Figure 4). Many receptor-like protein kinase genes were specifically upregulated in response to BBWV2-PAP1 infection (Figure 4B). In plants, most surface PRRs are receptor-like protein kinases in plants (Wang et al., 2016). The *Arabidopsis* leucine-rich repeat receptor kinases FLAGELLIN SENSING 2 and EF-TU RECEPTOR, which can recognize bacterial flagellin and EF-Tu, respectively, are well-characterized PRRs (Zipfel, 2009; Tang et al., 2017). PAMP perception upregulates a number of receptor-like protein kinases, indicating that PTI increases the capacity for pathogen recognition (Zipfel et al., 2004, 2006).

Broad bean wilt virus 2-PAP1 infection also resulted in the upregulation of various PTI signaling genes, including *mitogen-activated protein kinase* (*CA.*PGAv.1.6.scaffold200.11), *WRKY transcription factor 70* (*CA.*PGAv.1.6.scaffold788.4), and *WRKY transcription factor 70-like* (*CA.*PGAv.1.6.scaffold1018.1; Figure 4C), which can lead to changes in the transcription of numerous genes involved in defense responses (Wang et al., 2016; Peng et al., 2018). Various pathogenesis-related protein genes were also upregulated in BBWV2-PAP1-infected pepper (Figure 4D). The *basic pathogenesis-related protein 1* gene (*CA.*PGAv.1.6.scaffold608.26) was highly upregulated in an incompatible interaction with *Xanthomonas campestris* pv. *vesicatoria* in pepper (Kim and Hwang, 2000). Interestingly, it was demonstrated that the upregulation of the *basic pathogenesis-related protein 1* gene was intrinsically associated with increased ethylene production (Kim and Hwang, 2000). In pepper, treatment with aminoethoxyvinylglycine (AVG), an inhibitor of ethylene biosynthesis, resulted in the suppression of *basic pathogenesis-related protein 1* gene expression, indicating that ethylene is an upstream regulator of this gene (Kim and Hwang, 2000). In addition, other ethylene-responsive pathogenesis-related protein genes, i.e., *pathogenesis-related protein 2* and *pathogenesis-related protein 3*, which encode β-1,3-glucanases and chitinase, respectively (van Loon et al., 2006b; Mazarei et al., 2007), were specifically upregulated in response to infection with BBWV2-PAP1 (Figure 4D). Therefore, our results suggest that PTI responses triggered by BBWV2-PAP1 infection are associated with ethylene signaling in pepper. Indeed, ethylene production and the pathway genes were significantly upregulated in pepper infected with BBWV2-PAP1 (Figures 4E, 5B).

Ethylene is one of the important regulating factors in disease responses as well as growth and development in plants (van Loon et al., 2006a; Huang et al., 2016). Accumulating evidence suggests that the involvement of ethylene signaling in defense responses is linked to the activation of ROS production (Mersmann et al., 2010; Yang et al., 2017). Similarly, BBWV2-PAP1 infection elevated ROS accumulation as well as ethylene production in pepper (Figure 5). In defense responses, ROS can function as molecular signals to activate programmed cell death, thereby increasing symptom severity (Peng et al., 2018). Ethylene signaling has also been shown to positively regulate the FLS2 receptor accumulation in *Arabidopsis* (Mersmann et al., 2010), which is consistent with our observation that various receptor-like protein kinase genes were upregulated by BBWV2-PAP1 infection (Figure 4A). Ethylene signaling involves plant-specific transcription factors, such as ERFs, which regulate various responses to environmental stimuli (Muller and Munne-Bosch, 2015). While various ERFs have been well characterized recently, ERF5 was shown to activate ethylene-dependent defense genes, and its overexpression resulted in increased resistance to the necrotrophic pathogen *Botrytis cinerea* (Moffat et al., 2012). However, no visible phenotypic alterations were observed in transgenic tobacco plants that overexpress ERF5 (Fischer and Dröge-Laser, 2004). In this study, we demonstrated that overexpression of ERF5 can strongly enhance symptom severity in pepper even when infected with the mild BBWV2 RP1 strain (Figure 6). This further demonstrates that the ethylene signaling pathway is associated with the enhancement of symptom severity in BBWV2 infection.

A previous study showed that tobacco mosaic virus coat protein can be recognized as a PAMP to activate PTI and oxidative burst (Allan et al., 2001). Furthermore, for BBWV2, viral strain-specific MP was identified as a symptom severity determinant (Seo et al., 2017). In this study, we found that the viral strain-specific activation of PTI was associated with an increase in symptom severity in BBWV2 infection, that is, BBWV2 MP acts as a PAMP in a virus strain-specific manner. In this regard, it is interesting to consider how plant virus MP activates PTI because, in general, PTI is activated upon the extracellular recognition of PAMPs by PRRs. BBWV2 MP is localized to plasmodesmata (PD), and forms tubule structures to facilitate virus cell-to-cell movement (Xie et al., 2016). PD are plasma membrane-lined channels that provide symplastic continuity between neighboring cells (Maule et al., 2011). PD membranes also contain various receptor proteins and receptor-like protein kinases (Faulkner, 2013). A previous study revealed that the interactions between viral MP and PD-located receptor-like proteins are crucial for tubule formation and virus cell-to-cell movement (Amari et al., 2010). Therefore, it is possible that BBWV2 MP may interact with PD-located PRRs, resulting in PTI signaling activation.

Disease symptom development is a complex physiological process involving large transcriptomic changes. Thus, analysis of the DEGs in plant–virus interactions is important to explore the molecular basis of various physiological phenomena in plants. The transcriptional responses in pepper were notably different after infection with the two different BBWV2 strains (PAP1 and RP1), which had different symptoms. Our comparative transcriptome analysis revealed molecular genetic clues for the increase in symptom severity upon infection with BBWV2-PAP1. Overall, our findings improve understanding of molecular mechanisms underlying disease symptom development in plants and provide a basis for the future exploration of the functions of pepper genes in fundamental plant physiology.

## Data Availability Statement

The original contributions presented in the study are publicly available. This data can be found here: National Center for Biotechnology Information (NCBI) BioProject database under accession number PRJNA751625.

## Author Contributions

J-KS designed the experiments and supervised the project. S-JH, S-JK, BC, M-HK, and H-RK performed the experiments. S-JH, S-JK, and J-KS analyzed the data and wrote and revised the manuscript. All authors contributed to the article and approved the submitted version.

## Funding

This research was supported by grants from Agenda Program (PJ014878), and funded by the Rural Development Administration of Korea and Basic Science Research Program (NRF-2020R1I1A1A01072564) and the National Research Foundation of Korea. M-HK was supported by a graduate research fellowship from the Ministry of Education through the Brain Korea 21 Project (Global Smart Farm Division for Educating Innovative Human Resources).

## Conflict of Interest

The authors declare that the research was conducted in the absence of any commercial or financial relationships that could be construed as a potential conflict of interest.

## Publisher’s Note

Publisher’s Note: All claims expressed in this article are solely those of the authors and do not necessarily represent those of their affiliated organizations, or those of the publisher, the editors and the reviewers. Any product that may be evaluated in this article, or claim that may be made by its manufacturer, is not guaranteed or endorsed by the publisher.

## Supplementary Material

The Supplementary Material for this article can be found online at: https://www.frontiersin.org/articles/10.3389/fpls.2021.746543/full#supplementary-material

Supplementary Table S1 Primers used in this study for quantitative real-time PCR.

Supplementary Table S2 Combined DEGs for infection with BBWV2-PAP1 or RP1.

Supplementary Table S3 GO terms for up-regulated DEGs in response to BBWV2-PAP1 infection.

Supplementary Table S4 Validation of RNA sequencing data by quantitative real-time PCR.

## Footnotes

^1^ http://peppergenome.snu.ac.kr/
