# Supplementary material for: Viral Strain-Specific Activation of Pathogen-Associated Molecular Pattern-Triggered Immunity Enhances Symptom Severity in Broad Bean Wilt Virus 2 Infection
Source: Front Plant Sci. 2021 Sep 21;12:746543. doi: 10.3389/fpls.2021.746543 (PMC8549444; doi:10.3389/fpls.2021.746543)
Supplement: Supplementary Table S1 — Primers used in this study for quantitative real-time PCR. [file Table_1.DOC]

Supplementary Table S1. Primers used in this study for quantitative real-time PCR.

| **Primer** | **Primer sequence (5’ to 3’)** | **Target gene** |
| --- | --- | --- |
| bPR-1-F | TCACGCCCAAAATTCACCTC | basic pathogenesis-related protein 1 |
| bPR-1-R | TCAGACACCCACAACTCCAC |
| grip22-F | CCCTCCTACTCCAACCAATAC | ripening-related protein grip22 |
| grip22-R | AACGTCCTGCAAACCATCC |
| RLK25-F | TCGTTGCCATTGCTGTTTC | cysteine-rich receptor-like protein kinase 25 |
| RLK25-R | ACATCCCCAAATCCACCTTC |
| GRP-F | GGGAAAAGCCACTGAATCAC | glycine-rich protein |
| GRP-R | GACCAACCATCCATTGGAAC |
| PR-3-F | ACGATGATAGATGCCCTGCC | pathogenesis-related protein 3 |
| PR-3-R | TGCCATAAAATCCGCTACCC |
| WRKY70-F | GGAGGAAATACGGGCAGAAG | WRKY transcription factor 70 |
| WRKY70-R | AGGTGGACATTCCAGCATAG |
| ACC-F | CTCCCAATCCAGAAGAATTTCC | ACC oxidase |
| ACC-R | TTGCCCAAAACACTAAGTCC |
| LRR-RPK-F | GACCCGAACCAACATTACTAC | LRR receptor-like serine/threonine-protein kinase |
| LRR-RPK-R | TGGAAAGATACAAGGACCTCAC |
| MAPK-F | CAACTCCCACAACATCCAC | mitogen-activated protein kinase |
| MAPK-R | AATCTGCTCTTCTCCTATCCC |
| ERF5-F | GGGAAAAGGGCAAGAGAAG | ethylene responsive factor 5 |
| ERF5-R | TGGTGATAATGGTGACAAAGG |
| ABC11-F | GCTATACTAGACGAACTCCAAC | ABC transporter B family member 11 |
| ABC11-R | CACATCCTCTAAACCAACTCC |
| LEC1-F | TTCCCTTTTATTTGCCAGCC | chitin-binding lectin 1-like |
| LEC1-R | CCACACCAACCATCAAAATTAC |
| UBI2-F | TACCCTTCACCTTGTCCTCC | ubiquitin2 |
| UBI2-R | GCCATCCTCCAACTGTTTTC |
| BBWV2-R1-RT-Fw | TCACAGGTTATGCCGCTTGT | BBWV2 RNA1 |
| BBWV2-R1-RT-Rv | TCACTCGTCCCAAGCTGTTC |
