# Supplementary material for: Viral Strain-Specific Activation of Pathogen-Associated Molecular Pattern-Triggered Immunity Enhances Symptom Severity in Broad Bean Wilt Virus 2 Infection
Source: Front Plant Sci. 2021 Sep 21;12:746543. doi: 10.3389/fpls.2021.746543 (PMC8549444; doi:10.3389/fpls.2021.746543)
Supplement: Supplementary Table S4 — Validation of RNA sequencing data by quantitative real-time PCR. [file Table_4.doc]

Supplementary Table S4. Validation of RNA sequencing data by quantitative real-time PCR

| **Gene** | **Seq. Description** | **Log2-fold change by RNA sequencing** | | **Log2-fold change by qRT-PCR** | |
| --- | --- | --- | --- | --- | --- |
|  |  | **PAP1** | **RP1** | **PAP1** | **RP1** |
| CA.PGAv.1.6.scaffold608.26 | basic pathogenesis-related protein 1 | 8.29 | 0.24 | 11.28±1.523 | 0.36±0.051 |
| CA.PGAv.1.6.scaffold631.48 | ripening-related protein grip22 | 5.82 | -0.34 | 4.48±0.327 | 0.12±0.032 |
| CA.PGAv.1.6.scaffold674.24 | cysteine-rich receptor-like protein kinase 25 | 5.59 | -0.15 | 3.84±0.413 | -0.21±0.029 |
| CA.PGAv.1.6.scaffold1405.6 | glycine-rich protein | 4.94 | 0.98 | 5.92±0.211 | 0.54±0.041 |
| CA.PGAv.1.6.scaffold890.65 | pathogenesis-related protein 3 | 4.60 | -0.69 | 5.16±0.125 | -0.13±0.048 |
| CA.PGAv.1.6.scaffold788.4 | WRKY transcription factor 70 | 4.31 | -0.79 | 4.16±0.515 | -0.29±0.036 |
| CA.PGAv.1.6.scaffold793.11 | ACC oxidase | 4.07 | -0.05 | 5.85±0.602 | 0.22±0.031 |
| CA.PGAv.1.6.scaffold1537.4 | LRR receptor-like serine/threonine-protein kinase | 3.82 | 0.17 | 3.27±0.267 | -0.11±0.184 |
| CA.PGAv.1.6.scaffold200.11 | mitogen-activated protein kinase | 1.39 | -0.38 | 2.35±0.328 | -0.14±0.041 |
| CA.PGAv.1.6.scaffold296.14 | ethylene responsive factor 5 | 1.22 | 0.19 | 2.14±0.295 | 0.17±0.028 |
| CA.PGAv.1.6.scaffold484.97 | ABC transporter B family member 11 | -1.43 | -2.02 | -1.18±0.051 | -1.62±0.141 |
| CA.PGAv.1.6.scaffold837.4 | chitin-binding lectin 1-like | -1.48 | -1.43 | -1.21±0.274 | -1.34±0.146 |
